# Cardiovascular Magnetic Resonance Detects Inflammatory Cardiomyopathy in Symptomatic Patients with Inflammatory Joint Diseases and a Normal Routine Workup

**DOI:** 10.3390/jcm11051428

**Published:** 2022-03-05

**Authors:** George Markousis-Mavrogenis, Maria Bonou, Vasiliki Vartela, Genovefa Kolovou, Aliki Venetsanopoulou, Theodora Markatseli, Anastasia Skalkou, Zoi Tziortzioti, Paraskevi Voulgari, Sophie I. Mavrogeni

**Affiliations:** 1 Onassis Cardiac Surgery Center, 17674 Athens, Greece; georgemm32@gmail.com (G.M.-M.); vasvartela@yahoo.gr (V.V.); genovefa@kolovou.com (G.K.); 2 Department of Cardiology, Laikon Hospital, 11527 Athens, Greece; bonou.maria@yahoo.com; 3 Rheumatology Department, University of Ioannina, 45110 Ioannina, Greece; alikivenetsanopoulou@yahoo.com (A.V.); markatseli_d@yahoo.gr (T.M.); skalkou.anastasia@gmail.com (A.S.); tziortziotizoi@gmail.com (Z.T.); pvoulgar@uoi.gr (P.V.)

**Keywords:** late gadolinium enhancement, echocardiography, C-reactive protein, T1 mapping, extracellular volume fraction, rheumatoid arthritis, ankylosing spondylitis, psoriatic arthritis

## Abstract

Background. Patients with inflammatory joint diseases (IJD) are more likely to develop cardiovascular disease compared with the general population. We hypothesized that cardiovascular magnetic resonance (CMR) could identify cardiac abnormalities in patients with IJD and atypical symptoms unexplained by routine clinical evaluation. Patients-Methods. A total of 51 consecutive patients with IJD (32 with rheumatoid arthritis, 10 with ankylosing spondylitis, and 9 with psoriatic arthritis) and normal clinical, electrocardiographic and echocardiographic workups, were referred for CMR evaluation due to atypical chest pain, shortness of breath, and/or palpitations. Their CMR findings were compared with those of 40 non-IJD controls who were referred for the same reason. All participants were examined using either a 1.5 T or 3.0 T CMR system. For T1/T2 mapping, comparisons were performed separately for each field strength. Results. Biventricular systolic function was similar between groups. In total, 25 (49%) patients with IJD vs. 0 (0%) controls had replacement-type myocardial fibrosis (*p* < 0.001). The T2 signal ratio, early/late gadolinium enhancement, and extracellular volume fraction were significantly higher in the IJD group. Native T1 mapping was significantly higher in patients with IJD independent of the MRI field strength (*p* < 0.001 for both). T2 mapping was significantly higher in patients with IJD compared with controls only in those examined using a 1.5 T MR system—52.0 (50.0, 55.0) vs. 37.0 (33.5, 39.5), *p* < 0.001. Conclusions. In patients with IJD and a mismatch between cardiac symptoms and routine non-invasive evaluation, CMR uniquely identified a significant proportion of patients with myocardial inflammation. A CMR examination should be considered in patients with IJD in similar clinical settings.

## 1. Introduction

Rheumatoid arthritis (RA), ankylosing spondylitis (AS), and psoriatic arthritis (PsA) are autoimmune rheumatic diseases with predominant joint involvement, otherwise known as inflammatory joint diseases (IJD), that confer an increased risk of cardiovascular disease (CVD) [1,2]. Atypical cardiac symptoms and subclinical myocardial dysfunction have been described in patients with IJD and a preserved left ventricular ejection fraction (LVEF), without the presence of comorbid traditional CVD risk factors [3]. Furthermore, the development of heart failure (HF) in patients with IJD is independent of traditional risk factors [1,2,3,4,5]. However, only recently has it been demonstrated that patients with RA are at an increased risk of non-ischemic HF, occurring during the early stages of disease progression [6]. Additionally, a multicenter study of patients with RA showed a low prevalence of both subclinical atherosclerosis and a history of CVD events in these patients [7]. However, ultrasonographic evidence of subclinical atherosclerotic markers, such as intima media thickness, was not identified in early RA, suggesting that atherosclerosis occurs later in the course of the disease [8]. In addition, subclinical impairment of myocardial and endothelial function was identified in patients with early PsA [9].

In everyday practice, the evaluation of immune activation and disease severity in patients with IJD is primarily based on findings at physical examination, combined with plasma levels of C-reactive protein (CRP). However, an analysis of the multi-ethnic study of atherosclerosis (MESA) cohort showed that the utility of high-sensitivity CRP (hsCRP) is modest for predicting atherosclerotic CVD [10].These findings emphasize the need for a non-invasive, highly reproducible diagnostic modality that could identify the presence of potential myocardial inflammation independent of blood inflammatory indices and atherosclerotic risk.

Cardiovascular magnetic resonance (CMR) is a non-invasive imaging modality that utilizes non-ionizing radiation. CMR has been successfully used for the evaluation of myocardial inflammation in various autoimmune rheumatic diseases, including RA [11,12,13]. The ability of CMR to characterize myocardial tissues with regard to the presence of edema and replacement fibrosis, the latter using gadolinium-based paramagnetic contrast agents, is of great value for guiding clinical and therapeutic decision making in patients with IJD [14,15]. Both myocardial edema and fibrosis are the result of various pathologic processes taking place in IJD, and their early detection can potentially prevent the development of HF by prompting the initiation of appropriate treatment [15]. The introduction of mapping techniques and extracellular volume fraction (ECV) quantification has further increased the diagnostic yield of CMR regarding the detection of diffuse edema/fibrosis in the myocardium [16,17,18]. However, ECV may be confounded by co-existing myocardial inflammation [19,20].

We hypothesized that CMR could provide important insights about myocardial involvement in symptomatic patients with IJD and an otherwise normal routine diagnostic workup. The aim of our study was to evaluate adult symptomatic patients with IJD using CMR, and to compare them with a group of symptomatic non-IJD controls, both with normal routine clinical, laboratory, electrocardiographic, and echocardiographic evaluation results.

## 2. Methods

### 2.1. Participants

A cohort of 51 consecutive patients with IJD (32 RA, 10 AS, 9 PsA) and a preserved LVEF (as assessed by echocardiography) were evaluated using CMR and retrospectively included for analysis in this study. All patients received a clinical referral for CMR by a rheumatologist and/or cardiologist based on the following criteria:

- symptoms of shortness of breath, palpitations, and/or chest pain during the previous 3–6 months, and
- an otherwise normal routine clinical, laboratory, electrocardiographic, and echocardiographic evaluation.

Of these, 22 patients with RA, 7 with PsA, and 5 with AS were evaluated using a 1.5 T system (n *=* 34 in total), while 10 with RA, 5 with AS, and 2 with PsA were evaluated using a 3.0 T system (n *=* 17 in total).

The 51 patients with IJD were compared with age- and sex-matched healthy controls who underwent a CMR examination due to the same aforementioned referral criteria (n *=* 20 for each CMR field strength; in total n *=* 40). All controls were required to have a normal CMR examination before being retrospectively included in this study. The ethics committee of the University of Ioannina, including I. Kastanioudakis, E. Kostanti, M. Kitsanou, M. Mpaltogianni, E. Bairaktari, N. Rarra, and S. Giannou, decided to allow the use of these clinical data for this retrospective clinical research study (decision number 767/7-10-2021). All patients and controls provided written informed consent for use of their data in this study.

### 2.2. Echocardiographic Evaluation

For all referred patients, the standard echocardiographic evaluation included both systolic and diastolic function evaluation, wall motion characterization, the measurement of atrial/ventricular dimensions, and valvular function evaluation.

### 2.3. Clinical Characteristics of Patients with IJD

In patients with IJD, disease activity was assessed with the disease activity score of 28 (DAS28), Bath Ankylosing Spondylitis Disease Activity Index (BASDAI) and disease activity score in psoriatic arthritis (DAPSA) scores for RA, AS, and PsA, respectively [20]. RA patients were under treatment with nonsteroidal anti-inflammatory drugs (NSAIDs), methotrexate, and hydroxychloroquine. AS patients were under treatment with NSAIDs and etanercept. Lastly, all patients with PsA were under treatment with NSAIDs and methotrexate. In five patients (9.8%) the diagnosis of IJD was established 3–6 months after the development of the aforementioned symptoms.

## 3. Methods

**Figure 1:**
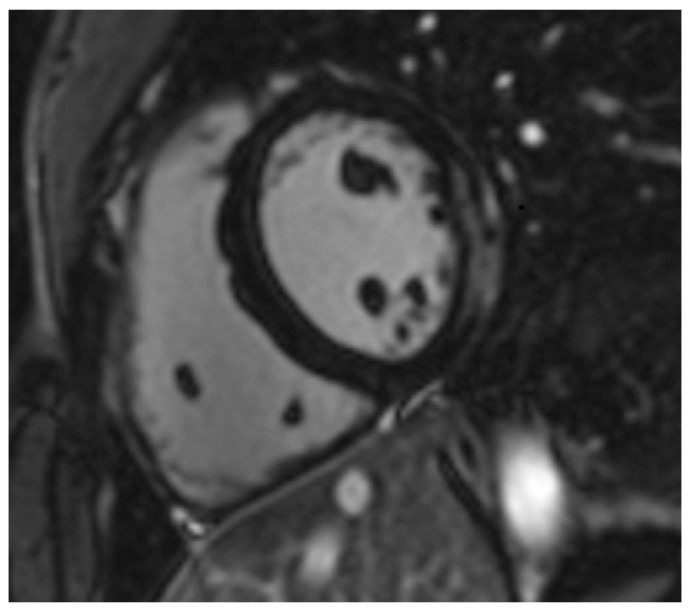
Short-axis inversion recovery image of a patient with ankylosing spondylitis showing evidence of subepicardial fibrosis (gray-white coloration) in the lateral wall.

CMR examinations were performed using a 1.5 T and 3.0 T scanners. The CMR protocol included standard steady-state free-precession cine CMR, black-blood T2-weighted short-tau inversion recovery images, T1-weighted spin-echo early gadolinium enhancement (EGE) images, and phase-sensitive inversion recovery late gadolinium enhancement (LGE) images, as described previously [14]. A dose of 0.1 mmol/kg gadobenate dimeglumine contrast medium was injected for EGE images and another 0.1 mmol/kg for LGE images (Figure 1), according to a previously published protocol [14]. This was carried out in all participants, seeing as none reported or exhibited adverse reactions to these contrast agents and had a normal renal function.

**Figure 2:**
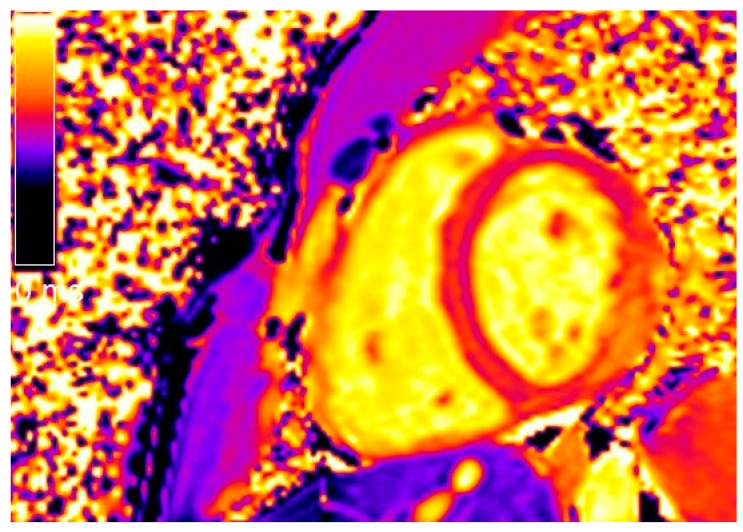
Short-axis native T1 mapping image of the same patient shown in Figure 1.

T1 mapping was performed using a modified Look-Locker inversion recovery (MOLLI) sequence with a 3(3)5 scheme on three representative short-axis positions, immediately before and 15 min. after contrast medium administration. T2 mapping was performed on three corresponding LV short axes using a black-blood prepared, navigator-gated, free-breathing hybrid gradient (echo planar imaging) and spin-echo multiecho sequences [16,17,18] (Figure 2).

### 3.1. CMR Data Analysis

Short-axis steady-state free-precession cine CMR was used to evaluate biventricular function (volumes and ejection fractions) according to a standardized protocol [15]. Global myocardial inflammation was assessed on T2-weighted images by calculating the T2 signal intensity ratio as the signal intensity of the myocardium divided by the signal intensity of skeletal muscle (T2 signal ratio) [15]. Global relative enhancement was calculated by measuring myocardial signal intensity on pre- and post-contrast T1-weighted spin-echo images relative to skeletal muscle [14]. The presence and pattern of non-ischemic LGE lesions were qualitatively assessed by consensus agreement of two experienced observers and expressed as a percentage of LV mass (%LGE). Native and post-contrast T1 mapping, ECV, and T2 mapping values were generated using dedicated plug-ins written for the OsiriX software, as described previously [21]. Global native/post-contrast myocardial T1, ECV, and T2 values were calculated as the mean value of three short-axis slices.

### 3.2. Statistical Analysis

Statistical analyses were performed using Stata SE v.16. The normality of continuous variables was determined by visual inspection of histograms and Q-Q plots. Normally distributed variables are presented as mean (standard deviation), not normally distributed continuous variables or interval variables are presented as median (interquartile range), and binary/categorical variables are presented as number (percentage). Between-group comparisons of normally distributed variables were carried out using independent-samples *t*-tests, those of not normally distributed continuous variables using Mann–Whitney U tests, and those of binary/categorical variables using chi-square tests. Correlations between CMR variables and disease duration in patients with IJD were examined using Spearman’s Rho test. Parametric CMR indices (native/post-contrast T1 mapping, T2 mapping) were examined separately for patients examined with 1.5 T and 3.0 T MRI systems to account for between-field differences in these variables. All other variables were examined collectively for both cases and controls. Statistical significance was considered for *p* ≤ 0.05.

## 4. Results

The baseline characteristics of the study cohort are presented in Table 1. Out of patients with IJD, 9 (18%) had a history of hypertension, 9 (18%) of hyperlipidemia, and 9 (18%) of diabetes mellitus. No patients had a family history of coronary artery disease. In addition, 6 (12%) were past smokers and 4 (8%) were current smokers. No significant differences were identified between the cases and controls regarding age and sex.

CMR findings are compared between cases and controls in Table 2 and Table 3. No significant differences between groups were identified in LV and right ventricular (RV) volumes and ejection fractions. LV mass was significantly lower in patients with IJD compared with controls—98.0 (65.0, 110.0) vs. 113.0 (103.5, 129.0), *p* < 0.001. Additional testing did not reveal any significant differences in median disease duration between patients with IJD that were below and above the median of LV mass—7.0 (3.0–10.0) vs. 7.0 (5.0–11.0), *p =* 0.381. In total, 25 (49%) patients with IJD had replacement-type myocardial fibrosis based on LGE positivity, in contrast to none of the healthy controls (*p* < 0.001). On average, the T2 signal ratio, EGE, LGE extent, and ECV were all significantly higher in the IJD group (Table 2). When utilizing locally used cut-off points for normal values of tissue characterization indices, none of the controls had pathologic values of EGE, LGE, or ECV, compared with 8 (16%) (*p =* 0.009), 25 (49%) (*p* < 0.001), and 22 (43%) (*p* < 0.001) patients with IJD, respectively (Table 2).

Comparisons of native/post-contrast T1 mapping and T2 mapping between controls and cases per MRI field strength are presented in Table 3. Native T1 mapping was significantly higher in patients with IJD compared with controls, independent of MRI field strength (*p* < 0.001 for both). Similarly, post-contrast T1 mapping was significantly lower in patients with IJD compared with controls, independent of MRI field strength (*p <* 0.01 for both). T2 mapping was significantly higher in patients with IJD compared with controls only in those examined with an MRI field strength of 1.5 T—52.0 (50.0, 55.0) vs. 37.0 (33.5, 39.5), *p* < 0.001, but not in those examined with an MRI field strength of 3.0 T (*p =* 0.10). No significant correlations were identified between any CMR indices and disease duration in patients with IJD (Table 4 and Table 5).

## 5. Discussion

In this study, we demonstrate that in a cohort of 51 patients with various types of IJD and shortness of breath, palpitations, and/or chest pain during the previous 3–6 months, along with an otherwise normal routine evaluation, CMR identified a considerable proportion of patients with cardiac abnormalities indicative of inflammatory cardiomyopathy. CMR findings appear to be independent of disease duration, and all patients had low circulating CRP levels, with an otherwise quiescent disease that was sufficiently treated with immunomodulatory medications.

Currently, data regarding the clinical use of CMR in patients with IJD is scarce, particularly for those presenting with unexplained symptoms such as those reported in our study. According to the literature, both symptomatic and asymptomatic patients with RA demonstrated higher native T1/T2 mapping and ECV values compared to controls, with the most significant differences identified in T2 mapping [22,23]. Our findings were also in agreement with previous studies supporting the presence of increased T1/T2 mapping, ECV, and reduced myocardial mass values in IJD [12,22,23,24,25]. Additionally, CMR was proven to be an important marker for disease monitoring in patients with AS and an abnormal transthoracic echocardiogram [25]. Thus, our findings further expand upon those of the previously mentioned studies to now include symptomatic patients with a normal echocardiographic evaluation.

It is important to note that the myocardial evaluation in our study was performed in symptomatic patients with a clinically quiescent IJD and an otherwise normal routine clinical/laboratory evaluation. In this context, CMR was the only diagnostic tool that demonstrated the presence of cardiac abnormalities suggestive of myocardial inflammation. Notably, all patients with IJD in this study had low circulating CRP levels (<10 mg/L), in spite of the abnormalities identified using CMR. Interestingly, in ~10% of RA cases with active disease, acute phase reactants may be within normal limits [26]. In addition, a previous analysis of the MESA cohort reported that there was no relationship between circulating CRP and native T1 mapping and ECV values in women [27]. In contrast, the same study reported that higher circulating CRP levels in men were associated only with higher T1 mapping, but not elevated ECV values [27]. This observation is noteworthy considering the fact that women are more likely than men to develop autoimmune disease, AS notwithstanding. However, the relationship of CMR indices with circulating CRP in patients with IJD and elevated CRP levels will need to be independently investigated in future studies.

Speckle-tracking echocardiography may show reduced global longitudinal LV and RV strain in patients with RA compared with healthy controls [28,29]. Nevertheless, evidence in patients with IJD is scarce. Echocardiography combines the advantages of low cost, broad availability, and expertise, but can be hampered by operator- and acoustic window-dependency. In addition, CMR offers the additional advantages of combined tissue characterization and functional assessment in a single examination, eliminating the issues of operator dependency and reliance on optimal acoustic windows.

An important finding of our study is that no included patients with IJD had any evidence of ischemic LGE lesions, even though the prevalence of coronary artery disease (CAD) in this population is considered high [30,31]. A potential explanation is that our patients were referred for CMR due to recent, but not acute, cardiac symptoms and a mismatch of symptoms with clinical/laboratory findings. In contrast, acute cases were presumably directly admitted to the hospital and catheterized immediately, thus precluding a CMR evaluation. An alternative explanation could be the relatively young age of the patient cohort, potentially leading to a reduced prevalence of symptomatic CAD.

Lastly, another point that merits discussion is the significantly lower LV mass that was identified in patients with IJD when compared with controls in our study. Such findings have also been reported in the past by our group in patients with antiphospholipid syndrome and systemic sclerosis with concomitant cardiac involvement [19,32], as well as in patients with RA by other researchers [33]. However, other studies have not been able to reproduce this finding [12]. Interestingly, a study utilizing echocardiography conversely demonstrated an increase in LV mass in patients with RA compared with healthy controls [32]. The apparent discordance of the echocardiography study could possibly be attributed to the potential limitations of LV mass estimation using echocardiography, such as acoustic window-dependency, particularly in women who constitute the majority of the study population. The same study also identified a significant association between LV mass and disease duration, a finding that was not evident in our study. The potential causes of decreases in LV mass in patients with IJD and the discordant findings of the echocardiography study have been discussed extensively in a previous publication [33].

Lastly, in our study population, no significant correlations between CMR indices and disease duration were identified. To our knowledge, except for the previously reported correlation of LV mass with disease duration in patients with RA [34], no other publications have reported any such associations until now, which is in agreement with our findings. Interestingly, we identified, on average, significantly higher T2 mapping values in patients with IJD compared with the controls in the 1.5 T comparison. This finding, however, was not significant in the 3.0 T comparison. We attribute this difference to chance inclusion of patients with a higher inflammatory burden in the 1.5 T analysis.

### Clinical Implications

Based on our findings, in patients with IJD with:

- otherwise optimally treated and quiescent IJD,
- symptoms of shortness of breath, palpitations, or chest pain and
- normal clinical, laboratory and echocardiographic findings,

CMR may be considered to rule out the presence of inflammatory cardiomyopathy. The findings of CMR can then be used to tailor individual therapeutic interventions with cardioprotective and immunomodulatory treatment.

## 6. Limitations

Our study has the following limitations:

1. a relatively small sample size.
2. a lack of CMR follow up in the majority of patients.
3. referral bias, because the CMR study was based only on clinical referrals.
4. a comparison between IJD patients with and without the referral symptoms using CMR was not possible.

## 7. Conclusions

A considerable proportion of patients with IJD and symptoms of chest pain, shortness of breath, and/or palpitations show CMR evidence of inflammatory cardiomyopathy, in spite of normal findings in routine clinical/laboratory/echocardiographic evaluations. Myocardial changes in these patients could not have been identified without a complementary CMR examination. As such, an integrated CMR evaluation should be utilized as an imaging biomarker in patients with IJD presenting with the aforementioned symptoms, even if their clinical/laboratory/echocardiographic evaluation is normal.

## Figures and Tables

**Table 1:** The baseline characteristics of the total cohort, stratified by controls and cases. * *p* ≤ 0.05.

Variable	Controls	Cases (IJD)	*p*-Value
**Demographics**			
Group Size	40	51	N/A
Age (years)	45.5 (13.3)	50.6 (14.5)	0.085
Female sex	28 (70%)	29 (57%)	0.20
MRI Field:			N/A
1.5 T	20 (50%)	34 (67%)	
3.0 T	20 (50%)	17 (33%)	
**Presenting Symptoms**			
Shortness of Breath	0 (0%)	28 (54.9%)	<0.001 *
Chest Pain	0 (0%)	36 (70.6%)	<0.001 *
Palpitations	0 (0%)	29 (56.9%)	<0.001 *
**Disease Characteristics**			
DAS28 (RA only)	N/A	3.8 (3.35–5.35)	N/A
DAPSA (PsA only)	N/A	8.9 (2.5–14.6)	N/A
BASDAI (AS only)	N/A	4.2 (3.8–4.4)	N/A
CRP (mg/L)	N/A	5.0 (3.5–6.0)	N/A
Disease Duration (years)	N/A	7.0 (5.0–10.0)	N/A
**Immunomodulatory Medications**			
NSAIDs	0 (0%)	51 (100%)	<0.001 *
Methotrexate	0 (0%)	41 (80.4%)	<0.001 *
Hydroxychloroquine	0 (0%)	32 (62.7%)	<0.001 *
Etanercept	0 (0%)	10 (19.6%)	<0.001 *

IJD—inflammatory joint disease; MRI—magnetic resonance imaging; DAS28—disease activity score-28; RA—rheumatoid arthritis; DAPSA—disease activity score in psoriatic arthritis; PsA—psoriatic arthritis; BASDAI—Bath Ankylosing Spondylitis Disease Activity Index; AS—ankylosing spondylitis; CRP—C-reactive protein; NSAIDs—non-steroidal anti-inflammatory drugs.

**Table 2:** Comparison of CMR indices and ECV between controls and cases. * *p* ≤ 0.05.

Variable	Controls	Cases (IJD)	*p*-Value
Group Size	40	51	N/A
**Biventricular Function and Volumes**			
LVEDV (mL)	128.5 (107.0, 143.5)	133.0 (113.0, 156.0)	0.30
LVESV (mL)	52.0 (41.0, 59.5)	53.0 (39.0, 68.0)	0.73
LVEF (%)	61.0 (58.0, 64.5)	62.0 (58.0, 66.0)	0.55
LV Mass (g)	113.0 (103.5, 129.0)	98.0 (65.0, 110.0)	<0.001 *
RVEDV (mL)	107.5 (98.0, 144.5)	117.0 (95.0, 155.0)	0.51
RVESV (mL)	48.0 (37.5, 54.5)	49.0 (34.0, 62.0)	0.40
RVEF (%)	61.0 (56.5, 65.0)	61.0 (53.0, 64.0)	0.57
**Tissue Characterization Indices**			
T2 Signal Ratio	1.5 (1.3, 1.8)	1.7 (1.4, 2.0)	0.010 *
EGE	2.0 (1.6, 2.3)	2.3 (1.8, 3.6)	0.025 *
LGE (%)	0.0 (0.0, 0.0)	0.0 (0.0, 5.0)	<0.001 *
ECV (%)	26.0 (23.5, 26.0)	29.0 (25.0, 32.0)	<0.001 *
**Cut-off Points for Normal Values**			
T2 Signal Ratio > 1.9	6 (15%)	16 (31%)	0.070
EGE > 4	0 (0%)	8 (16%)	0.009 *
LGE (presence)	0 (0%)	25 (49%)	<0.001 *
ECV > 29%	0 (0%)	22 (43%)	<0.001 *

LVEDV—left ventricular end diastolic volume; LVESV—left ventricular end systolic volume; LVEF—left ventricular ejection fraction; RVEDV—right ventricular end diastolic volume; RVESV—right ventricular end systolic volume; RVEF—right ventricular ejection fraction; EGE—early gadolinium enhancement; LGE—late gadolinium enhancement; LGE (%)—late gadolinium enhancement as % of left ventricular mass; ECV—extracellular volume fraction.

**Table 3:** Comparison of T1 and T2 mapping between controls and cases per MRI field strength. * *p* ≤ 0.05.

Variable	Controls 1.5 T	IJD Cases 1.5 T	*p*-Value	Controls 3.0 T	IJD Cases 3.0 T	*p*-Value
Group Size	20	34	N/A	20	17	N/A
Native T1 Mapping	973.5 (963.5, 979.5)	1032.0 (994.0, 1081.0)	<0.001 *	1164.0 (1153.5, 1171.0)	1261.0 (1235.0, 1280.0)	<0.001 *
Post-Contrast T1 Mapping	431.0 (419.5, 451.0)	403.0 (350.0, 421.0)	0.002 *	500.5 (485.0, 524.0)	433.0 (381.0, 513.0)	0.009 *
T2 mapping	37.0 (33.5, 39.5)	52.0 (50.0, 55.0)	<0.001 *	44.0 (39.0, 48.0)	46.0 (42.0, 50.0)	0.10

CMR—cardiovascular magnetic resonance; MRI—magnetic resonance imaging; IJD—inflammatory joint disease.

**Table 4:** Spearman’s Rank-Order correlation analysis of relevant CMR findings contrasted with disease duration in years. Analyses include only IJD patients.

Variable	Spearman’s Rho	*p*-Value
LVEF	0.006	0.969
RVEF	−0.085	0.555
EGE	0.001	0.993
LGE (%)	0.091	0.527
T2 Signal Ratio	−0.214	0.132
LV Mass	0.121	0.398
ECV	−0.026	0.854

LVEF—left ventricular ejection fraction; RVEF—right ventricular ejection fraction; EGE—early gadolinium enhancement; LGE (%)—late gadolinium enhancement as % of left ventricular mass; ECV—extracellular volume fraction.

**Table 5:** Spearman’s Rank-Order correlation analysis of T1 and T2 mapping contrasted with disease duration in years (per CMR field strength). Analyses include only IJD patients.

Variable	1.5 T		3.0 T	
	Spearman’s Rho	*p*-Value	Spearman’s Rho	*p*-Value
Native T1 Mapping	0.294	0.091	0.074	0.778
T2 Mapping	0.003	0.985	0.214	0.409

## Data Availability

Data can be provided upon reasonable request.

## References

[1] Nicola P.J., Maradit-Kremers H., Roger V.L., Jacobsen S.J., Crowson C.S., Ballman K.v., Gabriel S.E. (2005). The Risk of Congestive Heart Failure in Rheumatoid Arthritis: A Population-Based Study over 46 Years. Arthritis Rheum..

[2] Myasoedova E., Crowson C.S., Nicola P.J., Maradit-Kremers H., Davis J.M., Roger V.L., Therneau T.M., Gabriel S.E. (2011). The Influence of Rheumatoid Arthritis Disease Characteristics on Heart Failure. J. Rheumatol..

[3] Crowson C.S., Nicola P.J., Kremers H.M., O’Fallon W.M., Therneau T.M., Jacobsen S.J., Roger V.L., Ballman K.V., Gabriel S.E. (2005). How Much of the Increased Incidence of Heart Failure in Rheumatoid Arthritis Is Attributable to Traditional Cardiovascular Risk Factors and Ischemic Heart Disease?. Arthritis Rheum..

[4] Løgstrup B.B., Ellingsen T., Pedersen A.B., Kjærsgaard A., Bøtker H.E., Maeng M. (2018). Development of Heart Failure in Patients with Rheumatoid Arthritis: A Danish Population-Based Study. Eur. J. Clin. Investig..

[5] Mantel Ä., Holmqvist M., Andersson D.C., Lund L.H., Askling J. (2017). Association Between Rheumatoid Arthritis and Risk of Ischemic and Nonischemic Heart Failure. J. Am. Coll. Cardiol..

[6] Ahlers M.J., Lowery B.D., Farber-Eger E., Wang T.J., Bradham W., Ormseth M.J., Chung C.P., Stein C.M., Gupta D.K. (2020). Heart Failure Risk Associated With Rheumatoid Arthritis-Related Chronic Inflammation. J. Am. Heart Assoc..

[7] Ruscitti P., Margiotta D.P.E., MacAluso F., Iacono D., D’Onofrio F., Emmi G., Atzeni F., Prete M., Perosa F., Sarzi-Puttini P. (2017). Subclinical Atherosclerosis and History of Cardiovascular Events in Italian Patients with Rheumatoid Arthritis: Results from a Cross-Sectional, Multicenter GIRRCS (Gruppo Italiano Di Ricerca in Reumatologia Clinica e Sperimentale) Study. Medicine.

[8] Gobbi C.A., Asbert P., Alba P.B., Resk J., Dotto G., Demarchi M., Cuvertino E., Pepe G.A., Salica D.A., Albiero E.H. (2019). Subclinical Markers of Atherosclerosis and Cardiovascular Risk Factors in Early Arthritis. Rev. Fac. Cienc. Med. Cordoba Argent..

[9] Gullo L.A., Rodríguez-Carrio J., Aragona C.O., Dattilo G., Zito C., Suárez A., Loddo S., Atteritano M., Saitta A., Mandraffino G. (2018). Subclinical Impairment of Myocardial and Endothelial Functionality in Very Early Psoriatic and Rheumatoid Arthritis Patients: Association with Vitamin D and Inflammation. Atherosclerosis.

[10] Cainzos-Achirica M., Miedema M.D., McEvoy J.W., Cushman M., Dardari Z., Greenland P., Nasir K., Budoff M.J., Al-Mallah M.H., Yeboah J. (2018). The Prognostic Value of High Sensitivity C-Reactive Protein in a Multi-Ethnic Population After More Than 10 Years of Follow-Up: The Multi-Ethnic Study of Atherosclerosis (MESA). Int. J. Cardiol..

[11] Kobayashi H., Kobayashi Y., Yokoe I., Akashi Y., Takei M., Giles J.T. (2017). Magnetic Resonance Imaging-Detected Myocardial Inflammation and Fibrosis in Rheumatoid Arthritis: Associations With Disease Characteristics and N-Terminal Pro-Brain Natriuretic Peptide Levels. Arthritis Care Res..

[12] Ntusi N.A.B., Francis J.M., Gumedze F., Karvounis H., Matthews P.M., Wordsworth P.B., Neubauer S., Karamitsos T.D. (2019). Cardiovascular Magnetic Resonance Characterization of Myocardial and Vascular Function in Rheumatoid Arthritis Patients. Hell. J. Cardiol..

[13] Mavrogeni S., Karabela G., Stavropoulos E., Gialafos E., Sfendouraki E., Kyrou L., Kolovou G. (2013). Imaging Patterns of Heart Failure in Rheumatoid Arthritis Evaluated by Cardiovascular Magnetic Resonance. Int. J. Cardiol..

[14] Friedrich M.G., Sechtem U., Schulz-Menger J., Holmvang G., Alakija P., Cooper L.T., White J.A., Abdel-Aty H., Gutberlet M., Prasad S. (2009). Cardiovascular Magnetic Resonance in Myocarditis: A JACC White Paper. J. Am. Coll. Cardiol..

[15] Mavrogeni S.I., Kitas G.D., Dimitroulas T., Sfikakis P.P., Seo P., Gabriel S., Patel A.R., Gargani L., Bombardieri S., Matucci-Cerinic M. (2016). Cardiovascular Magnetic Resonance in Rheumatology: Current Status and Recommendations for Use. Int. J. Cardiol..

[16] Ferreira V.M., Piechnik S.K., Dallarmellina E., Karamitsos T.D., Francis J.M., Choudhury R.P., Friedrich M.G., Robson M.D., Neubauer S. (2012). Non-Contrast T1-Mapping Detects Acute Myocardial Edema with High Diagnostic Accuracy: A Comparison to T2-Weighted Cardiovascular Magnetic Resonance. J. Cardiovasc. Magn. Reson..

[17] Won S., Davies-Venn C., Liu S., Bluemke D.A. (2013). Noninvasive Imaging of Myocardial Extracellular Matrix for Assessment of Fibrosis. Curr. Opin. Cardiol..

[18] Mavrogeni S., Apostolou D., Argyriou P., Velitsista S., Papa L., Efentakis S., Vernardos E., Kanoupaki M., Kanoupakis G., Manginas A. (2017). T1 and T2 Mapping in Cardiology: “Mapping the Obscure Object of Desire”. Cardiology.

[19] Markousis-Mavrogenis G., Koutsogeorgopoulou L., Katsifis G., Dimitroulas T., Kolovou G., Kitas G.D., Sfikakis P.P., Mavrogeni S.I. (2020). The Double-Edged Sword of T1-Mapping in Systemic Sclerosis—A Comparison with Infectious Myocarditis Using Cardiovascular Magnetic Resonance. Diagnostics.

[20] Lurz J.A., Luecke C., Lang D., Besler C., Rommel K.P., Klingel K., Kandolf R., Adams V., Schöne K., Hindricks G. (2018). CMR-Derived Extracellular Volume Fraction as a Marker for Myocardial Fibrosis: The Importance of Coexisting Myocardial Inflammation. JACC. Cardiovasc. Imaging.

[21] Mavrogeni S.I., Sfikakis P.P., Markousis-Mavrogenis G., Bournia V.K., Poulos G., Koutsogeorgopoulou L., Karabela G., Stavropoulos E., Katsifis G., Boki K. (2019). Cardiovascular Magnetic Resonance Imaging Pattern in Patients with Autoimmune Rheumatic Diseases and Ventricular Tachycardia with Preserved Ejection Fraction. Int. J. Cardiol..

[22] Ntusi N.A.B., Piechnik S.K., Francis J.M., Ferreira V.M., Matthews P.M., Robson M.D., Wordsworth P.B., Neubauer S., Karamitsos T.D. (2015). Diffuse Myocardial Fibrosis and Inflammation in Rheumatoid Arthritis: Insights From CMR T1 Mapping. JACC. Cardiovasc. Imaging.

[23] Greulich S., Mayr A., Kitterer D., Latus J., Henes J., Vecchio F., Kaesemann P., Patrascu A., Greiser A., Groeninger S. (2017). Advanced Myocardial Tissue Characterisation by a Multi-Component CMR Protocol in Patients with Rheumatoid Arthritis. Eur. Radiol..

[24] Ntusi N.A.B., Francis J.M., Sever E., Liu A., Piechnik S.K., Ferreira V.M., Matthews P.M., Robson M.D., Wordsworth P.B., Neubauer S. (2018). Anti-TNF Modulation Reduces Myocardial Inflammation and Improves Cardiovascular Function in Systemic Rheumatic Diseases. Int. J. Cardiol..

[25] Biesbroek P.S., Heslinga S.C., Konings T.C., van der Horst-Bruinsma I.E., Hofman M.B.M., van de Ven P.M., Kamp O., van Halm V.P., Peters M.J.L., Smulders Y.M. (2017). Insights into Cardiac Involvement in Ankylosing Spondylitis from Cardiovascular Magnetic Resonance. Heart.

[26] Neto N.S.R., de Carvalho J.F. (2009). The Use of Inflammatory Laboratory Tests in Rheumatology. Rev. Bras. De Reumatol..

[27] Marques M.D., Nauffal V., Ambale-Venkatesh B., Vasconcellos H.D., Wu C., Bahrami H., Tracy R.P., Cushman M., Bluemke D.A., Lima J.A.C. (2018). Association Between Inflammatory Markers and Myocardial Fibrosis. Hypertension.

[28] Fine N.M., Crowson C.S., Lin G., Oh J.K., Villarraga H.R., Gabriel S.E. (2014). Evaluation of myocardial function in patients with rheumatoid arthritis using strain imaging by speckle-tracking echocardiography. Ann. Rheum. Dis..

[29] Naseem M., Samir S., Ibrahim I.K., Khedr L., Shahba A.A.E. (2019). 2-D speckle-tracking assessment of left and right ventricular function in rheumatoid arthritis patients with and without disease activity. J. Saudi Hear. Assoc..

[30] Milwidsky A., Ziv-Baran T., Letourneau-Shesaf S., Keren G., Taieb P., Berliner S., Shacham Y. (2017). CRP Velocity and Short-Term Mortality in ST Segment Elevation Myocardial Infarction. Biomarkers.

[31] Hansen P.R., Feineis M., Abdulla J. (2019). Rheumatoid Arthritis Patients Have Higher Prevalence and Burden of Asymptomatic Coronary Artery Disease Assessed by Coronary Computed Tomography: A Systematic Literature Review and Meta-Analysis. Eur. J. Intern. Med..

[32] Mavrogeni S.I., Markousis-Mavrogenis G., Karapanagiotou O., Toutouzas K., Argyriou P., Velitsista S., Kanoupakis G., Apostolou D., Hautemann D., Sfikakis P.P. (2019). Silent Myocardial Perfusion Abnormalities Detected by Stress Cardiovascular Magnetic Resonance in Antiphospholipid Syndrome: A Case-Control Study. J. Clin. Med..

[33] Giles J.T., Malayeri A.A., Fernandes V., Post W., Blumenthal R.S., Bluemke D., Vogel-Claussen J., Szklo M., Petri M., Gelber A.C. (2010). Left Ventricular Structure and Function in Patients with Rheumatoid Arthritis, as Assessed by Cardiac Magnetic Resonance Imaging. Arthritis Rheum..

[34] Rudominer R.L., Roman M.J., Devereux R.B., Paget S.A., Schwartz J.E., Lockshin M.D., Crow M.K., Sammaritano L., Levine D.M., Salmon J.E. (2009). Independent Association of Rheumatoid Arthritis with Increased Left Ventricular Mass but Not with Reduced Ejection Fraction. Arthritis Rheum..

